# Regulation of SARS CoV-2 host factors in the kidney and heart in rats with 5/6 nephrectomy—effects of salt, ARB, DPP4 inhibitor and SGLT2 blocker

**DOI:** 10.1186/s12882-022-02747-1

**Published:** 2022-03-24

**Authors:** Yingquan Xiong, Denis Delic, Shufei Zeng, Xin Chen, Chang Chu, Ahmed A. Hasan, Bernhard K. Krämer, Thomas Klein, Lianghong Yin, Berthold Hocher

**Affiliations:** 1 Fifth Department of Medicine (Nephrology/Endocrinology/Rheumatology), University Medical Centre Mannheim, University of Heidelberg, Mannheim, Germany; 2 Department of Nephrology, Charité - Universitätsmedizin Berlin, Campus Mitte, Berlin, Germany; 3 Boehringer Ingelheim Pharma GmbH & Co. KG, Biberach, Germany; 4 Division of Nephrology, Nanfang Hospital, Southern Medical University, Guangzhou, China; 5 Department of Nephrology, the First Affiliated Hospital of Jinan University, Guangzhou, China; 6 Institute of Nutritional Sciences, University of Potsdam, Potsdam, Germany; 7 Institute of Pharmacy, Free University of Berlin, Berlin, Germany; 8 Reproductive and Genetic Hospital of CITIC-Xiangya, Changsha, China; 9 Key Laboratory of Study and Discovery of Small Targeted Molecules of Hunan Province, School of Medicine, Hunan Normal University, Changsha, China; 10 Institute of Medical Diagnostics, IMD, Berlin, Berlin, Germany

**Keywords:** SARS CoV-2 host factors, 5/6 nephrectomy, High-salt diet, ARB, DPP4 inhibitor, SGLT2 blocker

## Abstract

**Background:**

Host factors such as angiotensin-converting enzyme 2 (ACE2) and the transmembrane protease, serine-subtype-2 (TMPRSS2) are important factors for SARS-CoV-2 infection. Clinical and pre-clinical studies demonstrated that RAAS-blocking agents can be safely used during a SARS-CoV-2 infection but it is unknown if DPP-4 inhibitors or SGLT2-blockers may promote COVID-19 by increasing the host viral entry enzymes ACE2 and TMPRSS2.

**Methods:**

We investigated telmisartan, linagliptin and empagliflozin induced effects on renal and cardiac expression of ACE2, TMPRSS2 and key enzymes involved in RAAS (REN, AGTR2, AGT) under high-salt conditions in a non-diabetic experimental 5/6 nephrectomy (5/6 Nx) model. In the present study, the gene expression of *Ace2*, *Tmprss2*, *Ren*, *Agtr2* and *Agt* was assessed with qRT-PCR and the protein expression of ACE2 and TMPRSS2 with immunohistochemistry in the following experimental groups: Sham + normal diet (ND) + placebo (PBO); 5/6Nx + ND + PBO; 5/6Nx + high salt-diet (HSD) + PBO; 5/6Nx + HSD + telmisartan; 5/6Nx + HSD + linagliptin; 5/6Nx + HSD + empagliflozin.

**Results:**

In the kidney, the expression of *Ace2* was not altered on mRNA level under disease and treatment conditions. The renal TMPRSS2 levels (mRNA and protein) were not affected, whereas the cardiac level was significantly increased in 5/6Nx rats. Intriguingly, the elevated TMPRSS2 protein expression in the heart was significantly normalized after treatment with telmisartan, linagliptin and empagliflozin.

**Conclusions:**

Our study indicated that there is no upregulation regarding host factors potentially promoting SARS-CoV-2 virus entry into host cells when the SGLT2-blocker empagliflozin, telmisartan and the DPP4-inhibitor blocker linagliptin are used. The results obtained in a preclinical, experimental non-diabetic kidney failure model need confirmation in ongoing interventional clinical trials.

## Background

Cardiovascular and renal diseases are considered as risk factors for increased coronavirus disease 2019 (COVID-19) disease severity and worse outcomes, including higher mortality. During the COVID-19 pandemic, tight control of glucose levels and prevention of complications associated with diabetes might be crucial in patients with diabetes to lower the susceptibility and severe course of COVID-19. Recent studies suggest that drugs interfering with the renin–angiotensin–aldosterone system (RAAS) or dipeptidyl peptidase 4 (DPP4) inhibitors can be used safely in patients with diabetes mellitus and COVID-19 [1–3]. In addition, the use of sodium-glucose cotransporter 2 (SGLT2) blockers seems to be a promising adjunct treatment option in patients with SARS-CoV2 infection and type 2 diabetes mellitus (T2DM) whereas an increased risk of protracted ketonemia and diabetic ketoacidosis was also reported [4].

ACE2 plays a central role in the regulation of RAAS and is involved in cardiac function, the development of hypertension and diabetes mellitus [5]. ACE2 exerts its protective effects by converting pro-inflammatory and pro-hypertensive AngII into anti-inflammatory and anti-hypertensive Ang1-7. ACE2 has been identified as a receptor for coronaviruses, including SARS-CoV-2. Once attached to ACE2 through the binding with the receptor binding domain in the viral spike protein, it is primed by the host TMPRSS2, which can enhance this endocytic way of entry but is not essential [6, 7]. An alternative route of viral entry is the direct fusion of the viral envelope and the cell membrane which is ACE2- and TMPRSS2-independent [8]. Increased ACE2 expression was observed as a response to inflammation, heart failure, lung injury and fibrosis [9–12] which led to increased AngII level and might facilitate the viral entry. In contrast, AngII can induce the internalization and degradation of ACE2 in an AT1R-dependent manner [13]. In addition, MERS-CoV binds to human DPP4/CD26 to infect host cells [14] and a recent study predicts the structure of the SARS-CoV-2 spike glycoprotein and its glycan shield pattern suggests that DPP4/CD26 might be a receptor for SARS-CoV-2 [15] which needs further validation. The increased presence of ACE2 or DPP4 might contribute to increased disease severity of infected patients.

In experimental preclinical models, the effects of RAAS blocking drugs on cardiac and renal ACE2 mRNA and/or protein expression led to controversial results. *Ace2* mRNA expression was increased in the left ventricle of normotensive rats after lisinopril or losartan treatment [16] whereas no increase in *Ace2* mRNA level was observed after coronary artery ligation and treatment with valsartan, ramipril or both compared to control [17]. In kidneys, telmisartan treatment resulted in increased expression of renal *Ace2* mRNA expression [18]. No effects on renal *Ace2* and *Tmprss2* mRNA expression after telmisartan treatment were previously verified in an independent study [19]. In a recent study, it was shown that captopril and telmisartan both decrease kidney ACE2 protein in kidney membranes without significantly affecting protein abundance in total kidney lysates. Captopril significantly reduced ACE2 protein in kidney membranes while cytosolic ACE2 was increased [20]. Importantly, mice with comorbid diabetes (aging, high fat diet and streptozotocin-induced diabetes) are characterized by increased renal *Ace2* mRNA expression but not further affected after telmisartan treatment which led to the conclusion that the increased ACE2 level is a consequence of the comorbidity and not an effect after RAAS blockade [19].

Dietary salt intake is a known risk factor for hypertension and is associated with an imbalance of the RAAS. The high salt diet fed spontaneously hypertensive rats (SHR) showed slightly decreased cardiac *Ace2* mRNA and protein expression [21] and renal expression was attenuated in uni-nephrectomized rats with subsequent high salt diet intake [22] but the effects of RAAS blocking drugs in a salt-induced experimental model have not been investigated yet. Recognizing that people with chronic kidney disease, who are often consuming a high-salt diet and commonly prescribed RAAS blocking drugs and/or DPP4-inhibitor and/or SGLT2 blocker, are at increased risk of severe COVID-19 outcomes, we studied the expression profiles of ACE2 and TMPRSS2 and other genes involved in the RAAS in the kidney and the heart in a rat model that mimics this phenotype (impaired kidney function combined with a high salt intake – most patients consume unfortunately several times more salt than they actually should control blood pressure). Here we used the rat 5/6 nephrectomy model, one of the most well-established experimental non-diabetic CKD model which is characterized by increased hypertension, inflammation and fibrosis.

## Methods

### Animals

**Fig. 1:**
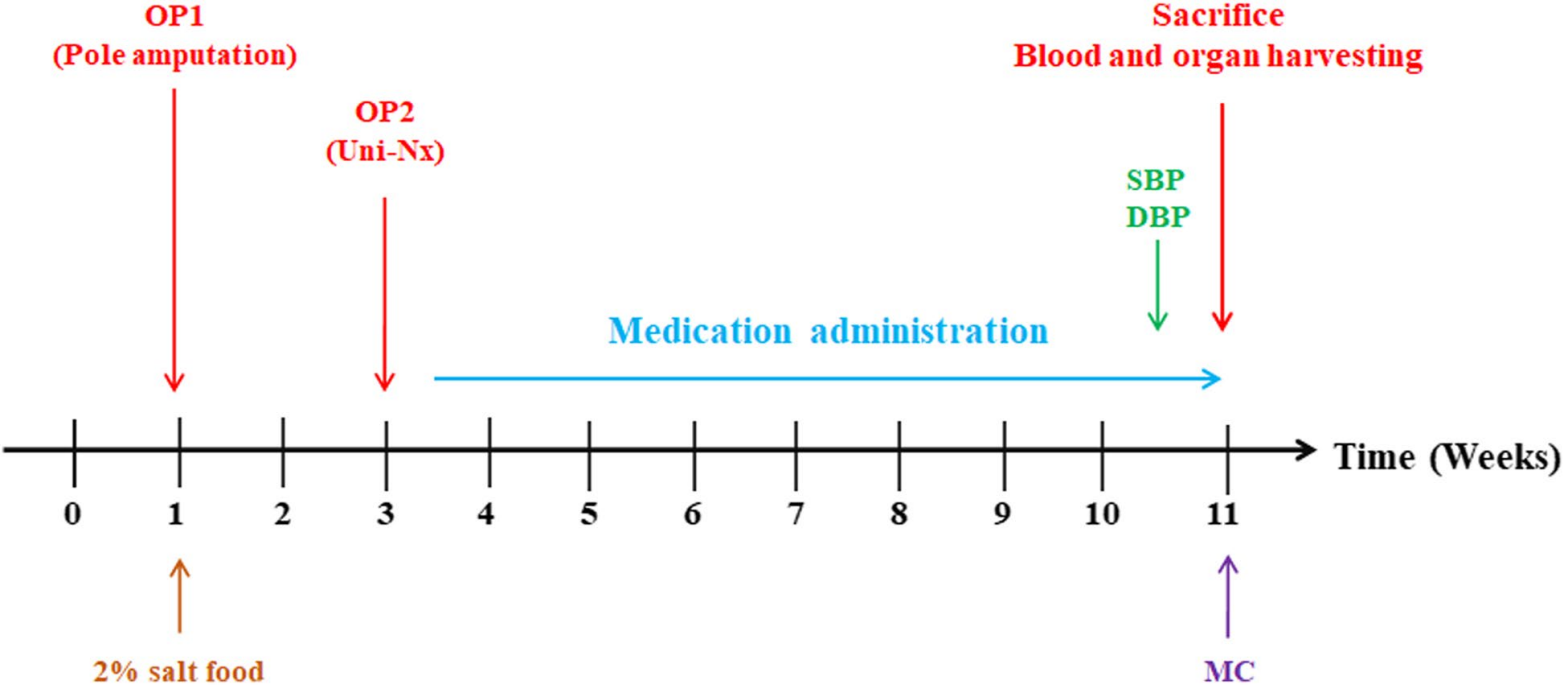
Time course of the animal study. *SBP* systolic blood pressure measurement, *DBP* diastolic blood pressure measurement; *MC* metabolic cages, *OP1* amputation of the poles of left kidney, *OP2* uninephrectomy on the right side, *Uni-Nx* unilaterally nephrectomized

The animal experiment was approved by the laboratory animal ethics committee (20,170,904,092,822, Jinan University, Guangzhou, China) following University Guidelines for Use of Laboratory Animals. A total of 91 male Wistar rats were assigned to the following groups: Sham + ND + PBO (*n* = 14); 5/6 Nx ND + PBO (*n* = 12); 5/6 Nx + HSD + PBO (*n* = 23); 5/6 Nx + HSD + telmisartan (5 mg/kg/day; *n* = 15); 5/6 Nx + HSD + linagliptin (3 mg/kg/day; *n* = 14); 5/6 Nx + HSD + empagliflozin (1.2 mg/kg/day; *n* = 13). The normal diet was standardized using AIN93M [23] and the high salt diet was adjusted to a 2% level of sodium chloride on this basis. The two feeds were produced under the codes LAD 3001 M and LAD0011HF2 (Trophic Animal Feed High-Tech Co., Ltd, China). The doses of telmisartan and linagliptin have been used in previous studies [24, 25]. Drug treatment via gavage was administered from week 3 until sacrifice (week 11). The rats were sacrificed at week 11 and plasma. Pentobarbital sodium (36–39 mg/kg body weight) was used to anesthetize the rats, which was administered intraperitoneally. Urine and perfused kidney and heart samples were collected and frozen until further analysis (Fig. 1). All experimental procedures (surgery, blood pressure measurements, metabolic cages, as well as plasma and urine analyses) were done as describe previously [26].

### Blood pressure measurement

Blood pressure was measured by non-invasive tail cuff plethysmography of the tail artery at week 11. The animal was placed in a restrainer, i.e. a tubular construction from which only the tail of the animal protruded. Then a blood pressure cuff and an electronic transducer were fixed to the tail of the animal. We waited until the animals were relaxed and got used to the restrainer. At intervals of 30 s, at least three measurements were taken to obtain reliable means of blood pressure. To get the animals used to this procedure, animals were trained before the actual measurement. The blood pressure diagrams and pulses were recorded and evaluated using the IITC Life Science tail cuff plethysmography blood pressure systems (IITC Life Science Inc., Woodland Hills, CA, USA).

### Biochemical evaluations

EDTA was added to blood samples followed by centrifugation (4,500 rpm) for 20 min at 4 ℃, then plasma was collected and stored at -20 ℃ until analysis. Urine samples were centrifuged (12,000 rpm) for 10 min at 4 ℃. The supernatant was frozen in liquid nitrogen until analysis. Levels of plasma creatinine, urea, glucose, and insulin as well as urinary creatinine, and total protein were detected using an automatic biochemistry analyzing system (Roche Cobas 6800, Roche Ltd, Switzerland). Levels of plasma BNP45 and urinary albumin were determined quantitatively using Rat BNP 45 ELISA Kit (Abcam, Cat#ab108816) and Rat Albumin ELISA Kit (Abcam, Cat#ab235642). The glomerular filtration rate-to-body weight ratio (GFR), albumin-to-creatinine ratio (ACR) were calculated. At a dose of 1 mg/day of empagliflozin urinary sodium and potassium excretion are not affected (data not shown).

### RNA isolation and quantitative real-time PCR (qRT-PCR)

Snap frozen kidney and heart tissues were homogenized with Precellys lysis with Precellys Steel 2.8 mm beads (PeqLab Biotechnology, Erlangen, Germany) and total RNA was isolated using the RNeasy Fibrous Tissue Mini Kit (QIAGEN, Hilden, Germany). Quality control and total RNA yield were quantified using the NanoDrop ND-1000 spectrophotometer (ThermoScientific, Wilmington, United States, DE). Renal and cardiac mRNA levels of Angiotensin I Converting Enzyme 2 (*Ace2*), Transmembrane Protease Serine Subtype 2 (*Tmprss2*), Renin (*Ren*), Angiotensin Receptor Type 2 (*Agtr2*) and Angiotensinogen (*Agt*) were analyzed by qRT- PCR on a SDS7900HT real-time PCR system (Applied Biosystems by ThermoFisher Scientific). Glyceraldehyde 3-phosphate dehydrogenase (*Gapdh*) was used as a housekeeping gene and experimental details were detailed previously [27]. All samples were run in duplicates and raw ct values were calculated using the SDS software v.2.4. All values were normalized to the mean expression level of the control group (Sham + ND + PBO) and the fold-change of expression compared to the control was calculated using the comparative Ct method (2-ΔΔct) [28].

### Immunohistochemistry

Kidney and cardiac tissue specimens were embedded in paraffin after fixation with 4% paraformaldehyde, cut into 3-μm paraffin sections for immunohistochemical staining. Sections were de-waxed twice using xylene and rehydrated with graded ethanol. After microwave antigen-retrieval, sections were blocked with 5% non-fat dry milk in phosphate-buffered saline/Tween 20 (PBS-T) for one hour and incubated respectively with primary antibodies specific to ACE2 (1:100 dilution; ab15348, Abcam, Cambridge, MA) and TMPRSS2 (1:50 dilution; EPR3861, ab92323, Abcam, Cambridge, MA) in 5% non-fat dry milk in PBS-T overnight at 4 °C. The sections were repeatedly washed 5 times with PBS-T, incubated with matching fluorescent secondary antibody (1:200, ab150075; Abcam) in PBS-T, and mounted with Fluoroshield mounting medium with 4′,6-diamidino-2-phenylindole (ab104139; Abcam). The fluorescent images were captured as described recently and analyzed using a computer-aided image analysis system as described previously [26].

### Statistical analysis

Statistical analyses were performed using GraphPad Prism 7 software (GraphPad, La Jolla, CA). The analysis of variance test followed by the Bonferroni post hoc test was applied for comparison of normally distributed data, and the data were presented as mean ± SEM. The Kruskal–Wallis test followed by Dunn’s post hoc test was used for non-normally distributed data, and the data were presented as median (25th—75th percentile), In all cases, differences were regarded as statistically significant if *P* < 0.05.

## Results

### Effects of salt, telmisartan, linagliptin and empagliflozin on clinical and biochemical parameters

At the end of the study high salt diet-fed placebo-treated 5/6 Nx rats (5/6 Nx + HSD + PBO) were characterized by significantly higher relative left kidney and relative heart weights, final systolic and diastolic blood pressures, final plasma creatinine, final urinary ACR level and final 24 h urinary protein excretion compared to normal-diet fed placebo-treated sham control rats (Table 1). In high salt diet-fed 5/6 Nx rats, treatment with telmisartan (5/6 Nx + HSD + TELM) significantly decreased the final body weight, final systolic and diastolic blood pressures versus 5/6 Nx + HSD + PBO rats (Table 1). Linagliptin treatment of high salt diet-fed 5/6 Nx rats (5/6 Nx + HSD + LINA) resulted in significantly decreased final body weight and final systolic blood pressure, whereby empagliflozin treatment led to significantly decreased relative liver weight compared to 5/6 Nx + HSD + PBO rats (Table 1).

**Table 1:** Clinical/Biochemical parameters

	**Sham + ND + PBO (*n* = 12–13)**	**5/6Nx + ND + PBO (*n* = 12–13)**	**5/6Nx + HSD + PBO (*n* = 15–23)**	**5/6Nx + HSD + TELM (*n* = 11–15)**	**5/6Nx + HSD + LINA (*n* = 13–15)**	**5/6Nx + HSD + EMPA (*n* = 10–11)**
Final body weight (g)	**475.35 ± 12.04**	**448.50 ± 17.38**	**443.35 ± 10.65**	**394.65 ± 12.51**^ab^	**382.39 ± 12.44**^ab^	**420.03 ± 11.68**^a^
Relative left kidney weight (mg/g)	**3.20(2.93–3.46)**^b^	**3.44(2.93–3.46)**	**3.64(3.31–4.35)**^a^	**3.56(3.15–4.26)**	**3.72(3.41–4.13)**^a^	**3.94(3.49–4.46)**^a^
Relative heart weight (mg/g)	**2.74 ± 0.06**^b^	**3.03 ± 0.08**	**3.74 ± 0.21**^a^	**3.97 ± 0.33**^a^	**3.77 ± 0.23**^a^	**3.58 ± 0.13**^a^
Relative liver weight (mg/g)	**24.03(23.27–25.62)**	**24.25(23.27–25.62)**	**26.46(22.36–27.54)**	**21.94(21.24–24.01)**	**22.88(21.80–24.56)**	**21.70(20.99–22.58)**^ab^
Final systolic blood pressure (mm Hg)	**124.66(118.33–130.50)**^b^	**153.66(118.33–130.50)**^a^	**153.00(149.00–163.66)**^a^	**127.33(118.00–129.66)**^b^	**133.83(129.75–142.25)**^b^	**126.33(124.66–131.00)**
Final diastolic blood pressure (mm Hg)	**101.56 ± 2.43**^b^	**122.77 ± 3.68**^a^	**123.61 ± 2,21**^a^	**99.36 ± 2.47**^b^	**116.52 ± 3.01**^a^	**98.63 ± 2.56**^b^
Final plasma creatinine (μmol/l)	**46.92 ± 0.76**^b^	**72.38 ± 2.27**	**84.78 ± 7.61**^a^	**102.80 ± 12.04**^a^	**95.71 ± 6.38**^a^	**88.80 ± 4.64**^a^
Final plasma urea (mmol/l)	**4.89 ± 0.21**	**11.18 ± 1.08**	**12.66 ± 3.46**	**16.94 ± 1.86**^a^	**15.84 ± 2.22**^a^	**14.87 ± 0.72**
Final plasma glucose (mmol)	**6.08(5.62–7.18)**	**9.03(5.62–7.18)**^**#**^	**6.52(5.36–7.69)**	**6.25(5.14–6.85)**	**7.16(6.20–8.51)**	**6.31(5.42–6.59)**
Final plasma insulin (μg/l)	**0.72(0.38–1.37)**	**0.53(0.38–1.37)**	**0.30(0.22–0.83)**	**0.20(0.10–0.33)**^a^	**0.19(0.13–0.51)**^a^	**0.18(0.09–0.32)**^a^
Final plasma BNP45 (ng/ml)	**2.23 ± 0.49**	**1.92 ± 0.30**	**2.13 ± 0.32**	**2.11 ± 0.32**	**2.15 ± 0.31**	**1.91 ± 0.41**
GFR/BW (ml/24 h/g)	**1.97(1.23–3.15)**	**1.62(1.23–3.15)**	**1.99(1.47–2.11)**	**1.47(1.22–1.72)**	**1.41(1.31–1.73)**	**1.74(1.34–2.15)**
Final urinary creatinine (mmol/l)	**11.41(6.39–15.23)**	**6.49(6.39–15.23)**	**6.74(4.64–8.69)**	**5.34(3.84–6.05)**^a^	**5.53(4.67–6.89)**^a^	**5.42(4.89–8.03)**
Final urinary ACR (mg/mmol)	**1.51(1.27–2.27)**^b^	**6.89(5.51–15.82)**^ab^	**38.49(11.10–282.90)**^a^	**44.10(2.57–249.2)**^a^	**92.80(20.31–219.70)**^a^	**34.87(9.45–230.80)**^a^
Final 24 h urinary protein excretion (mg/24 h)	**4.81(4.23–5.79)**^b^	**7.17(6.35–10.47)**^**b**^	**11.63(7.48–36.08)**^a^	**19.97(5.04–28.9)**^a^	**12.38(5.51–21.84)**^a^	**10.56(7.35–21.92)**^a^

GFR/BW (ml/24 h/g) = [urinary creatinine * urinary flow (ml/min)]/[serum creatinine * body weight]

Urinary ACR (mg/mmol) = urinary albuminuria / urinary creatinine

Normally distributed data were given as mean ± SEM. Non-normally distributed data were given as median (25th–75th percentile)

^#a^*p* < 0.05 vs. Sham + ND + PBO, ^*b^*p* < 0.05 vs. 5/6Nx + HSD + PBO

### Effects of salt, telmisartan, linagliptin and empagliflozin on renal and cardiac mRNA expression of genes associated with SARS-CoV-2 host factors and RAAS

In order to investigate the effects of salt, telmisartan, linagliptin and empagliflozin on the gene expression levels of the two key SARS-CoV-2 host factors *Ace2* and *Tmprss2* and genes involved in the RAAS, such as *Ren*, *Agtr2* and *Agt*, in the kidney and heart qRT-PCR was performed. Overall, the expression of *Ace2* was not affected in both kidney and heart in all experimental groups. Telmisartan and empagliflozin significantly increased the renal *Tmprss2* gene expression compared to 5/6 Nx + HSD + PBO rats whereas the cardiac *Tmprss2* expression was below the detection limit (Table 2). Importantly, telmisartan and empagliflozin increased *Tmprss2* mRNA levels are not significantly altered compared to the control group (Sham + ND + PBO).

**Table 2:** Renal and cardiac mRNA expression of SARS-CoV-2 host factors and genes involved in RAAS

	**Sham + ND + PBO (*n* = 6)**	**5/6Nx + ND + PBO (*n* = 6)**	**5/6Nx + HSD + PBO (*n* = 5–6)**	**5/6Nx + HSD + TELM (*n* = 6)**	**5/6Nx + HSD + LINA (*n* = 6)**	**5/6Nx + HSD + EMPA (*n* = 6)**
**mRNA expression (kidney)**						
*** Ace2***	**1.02 ± 0.09**	**1.19 ± 0.30**	**1.01 ± 0.33**	**1.14 ± 0.14**	**1.47 ± 0.47**	**1.33 ± 0.33**
*** Tmprss2***	**1.03 ± 0.12**	**0.50 ± 0.09**	**0.86 ± 0.09**	**2.11 ± 0.41**^b^	**0.97 ± 0.08**	**4.23 ± 2.92**^b^
*** Ren***	**1.06 ± 0.18**	**0.06 ± 0.02**^ab^	**0.06 ± 0.04**^a^	**1.56 ± 0.56**^b^	**0.12 ± 0.03**^a^	**2.52 ± 1.17**^b^
*** Agtr2***	**1.03 ± 0.13**	**0.78 ± 0.21**	**1.08 ± 0.19**	**1.02 ± 0.13**	**0.84 ± 0.16**	**3.09 ± 1.41**
*** Agt***	**1.10 ± 0.24**	**1.13 ± 0.28**	**1.02 ± 0.24**	**1.63 ± 0.29**	**1.57 ± 0.17**	**4.32 ± 1.67**
**mRNA expression (heart)**						
*** Ace2***	**1.04 ± 0.19**	**0.94 ± 0.06**	**1.17 ± 0.08**	**1.08 ± 0.08**	**0.77 ± 0.06**	**1.03 ± 0.08**
*** Tmprss2***	**n.d**	**n.d**	**n.d**	**n.d**	**n.d**	**n.d**
*** Ren***	**n.d**	**n.d**	**n.d**	**n.d**	**n.d**	**n.d**
*** Agtr2***	**1.01 ± 0.06**	**1.06 ± 0.07**	**1.13 ± 0.09**	**0.98 ± 0.06**	**0.91 ± 0.09**	**0.97 ± 0.5**
*** Agt***	**1.04 ± 0.12**	**1.79 ± 0.23**	**0.95 ± 0.04**	**1.19 ± 0.20**	**0.77 ± 0.09**	**0.92 ± 0.07**
	**Sham + ND + PBO (*n* = 12–14)**	**5/6Nx + ND + PBO (*n* = 7–12)**	**5/6Nx + HSD + PBO (*n* = 15–20)**	**5/6Nx + HSD + TELM (*n* = 7–11)**	**5/6Nx + HSD + LINA (*n* = 10–13)**	**5/6Nx + HSD + EMPA (*n* = 6–9)**
**protein expression**						
** ACE2 (kidney)**	**29.89**	**27.10**	**13.96**	**14.14**	**33.25**	**17.07**
	**(20.82–36.55)**	**(19.39–30.73)**	**(12.36–19.14)**^a^	**(9.35–19.34)**^a^	**(18.55–39.25)**^b^	**(12.59–19.96)**
** ACE2 (heart)**	**27.77**	**33.11**	**29.12**	**27.42**	**24.39**	**33.21**
	**(25.58–32.96)**	**(30.74–36.34)**	**(23.97–32.42)**	**(24.57–31.01)**	**(22.32–29.63)**	**(26.57–35.92)**
** TMPRSS2 (kidney)**	**13.44**	**13.88**	**12.77**	**12.35**	**12.21**	**11.60**
	**(11.64–17.31)**	**(11.89–15.73)**	**(8.53–14.21)**	**(10.79–18.26)**	**(9.72–13.90)**	**(10.62–13.29)**
** TMPRSS2 (heart)**	**11.90**	**29.57**	**25.23**	**10.40**	**7.85**	**12.00**
	**(10.63–15.00)**	**(25.12–33.16)**^a^	**(16.18–30.46)**^a^	**(8.60–18.57)**^b^	**(6.03–13.39)**^b^	**(8.59–16.27)**^b^

Normally distributed data were given as mean ± SEM. Non-normally distributed data were given as median × 10^6^ (25th–75th percentile × 10^6^). ^a^*p* < 0.05 vs. Sham + ND + PBO, ^b^*p* < 0.05 vs. 5/6Nx + HSD + PBO

Renal *Ren* expression was significantly decreased in 5/6 Nx + ND + PBO, 5/6 Nx + HSD + PBO and 5/6 Nx + HSD + LINA groups compared to the Sham + ND + PBO control group. Telmisartan and empagliflozin significantly normalized the renal expression of *Ren* versus 5/6 Nx + HSD + PBO rats (Table 2), whereas *Agtr2* and *Agt* were not significantly affected in any experimental groups (Table 2).

### Effects of salt, telmisartan, linagliptin and empagliflozin on renal and cardiac expression of proteins associated with SARS-CoV-2 host factors

**Fig. 2:**
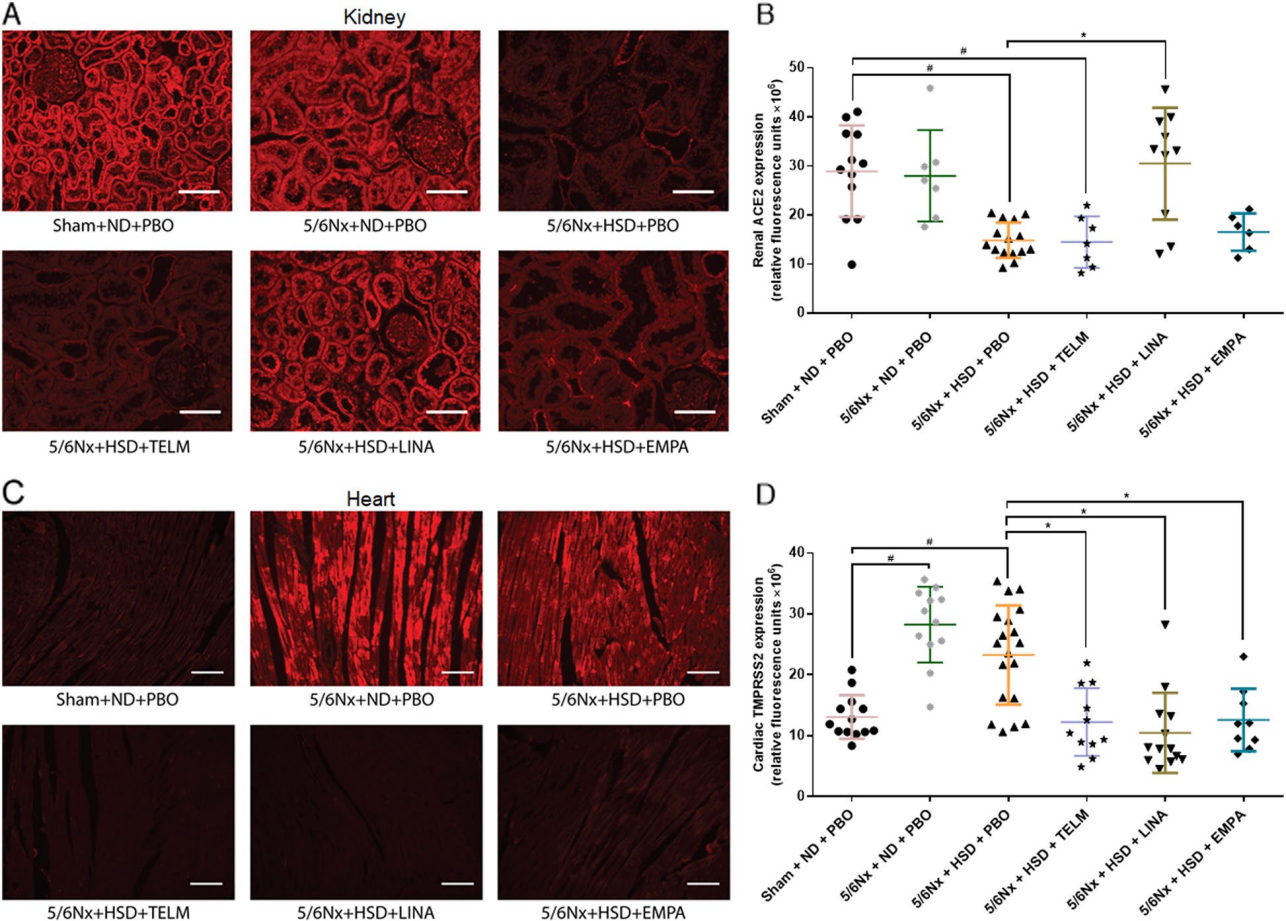
Renal ACE2 protein expression and cardiac TMPRSS2 protein expression in different groups. **A-B** Effects of high salt diet, telmisartan, linagliptin and empagliflozin on renal protein expression of ACE2 and cardiac protein expression of TMPRSS2. **A** Photomicrographs of immunofluorescence-stained kidneys. The red color indicates ACE2. **B** Renal protein expression of ACE2. **C** Photomicrographs of immunofluorescence-stained hearts. The red color indicates TMPRSS2. **D** Cardiac protein expression of ACE2. Magnification × 20 [scale bars = 100 μm]. ^#^*p* < 0.05 vs. Sham + ND + PBO, ^*^p < 0.05 vs. 5/6Nx + HSD + PBO

In the next step we examined the ACE2 and TMPRSS2 protein expressions in the kidney and heart using polyclonal ACE2 and TMPRSS2 antibodies as described previously [19]. The renal ACE2 protein expression was significantly decreased in the placebo or telmisartan treated high-salt diet fed 5/6 Nx rats compared to Sham + ND + PBO rats whereby linagliptin significantly increased the ACE2 protein levels in 5/6 Nx + HSD rats (Fig. 2A, B, Table 2) characterized by normalized ACE2 protein levels compared to Sham + ND + PBO or 5/6 Nx + ND + PBO rats (Fig. 2A, B, Table 2). In the corresponding heart tissues, there was no major change in ACE2 protein levels in all experimental groups (Table 2).

In kidneys the TMPRSS2 protein level was not significantly altered by the respective treatments. In contrast, in 5/6 Nx + ND + PBO and 5/6 Nx + HSD + PBO rats the cardiac TMPRSS2 expression was significantly increased compared to Sham + ND + PBO control rats (Fig. 2C, D, Table 2). Notably, telmisartan, linagliptin and empagliflozin normalized the increased cardiac TMPRSS2 level compared to 5/6 Nx + HSD + PBO rats (Fig. 2C, D, Table 2).

**Fig. 3:**
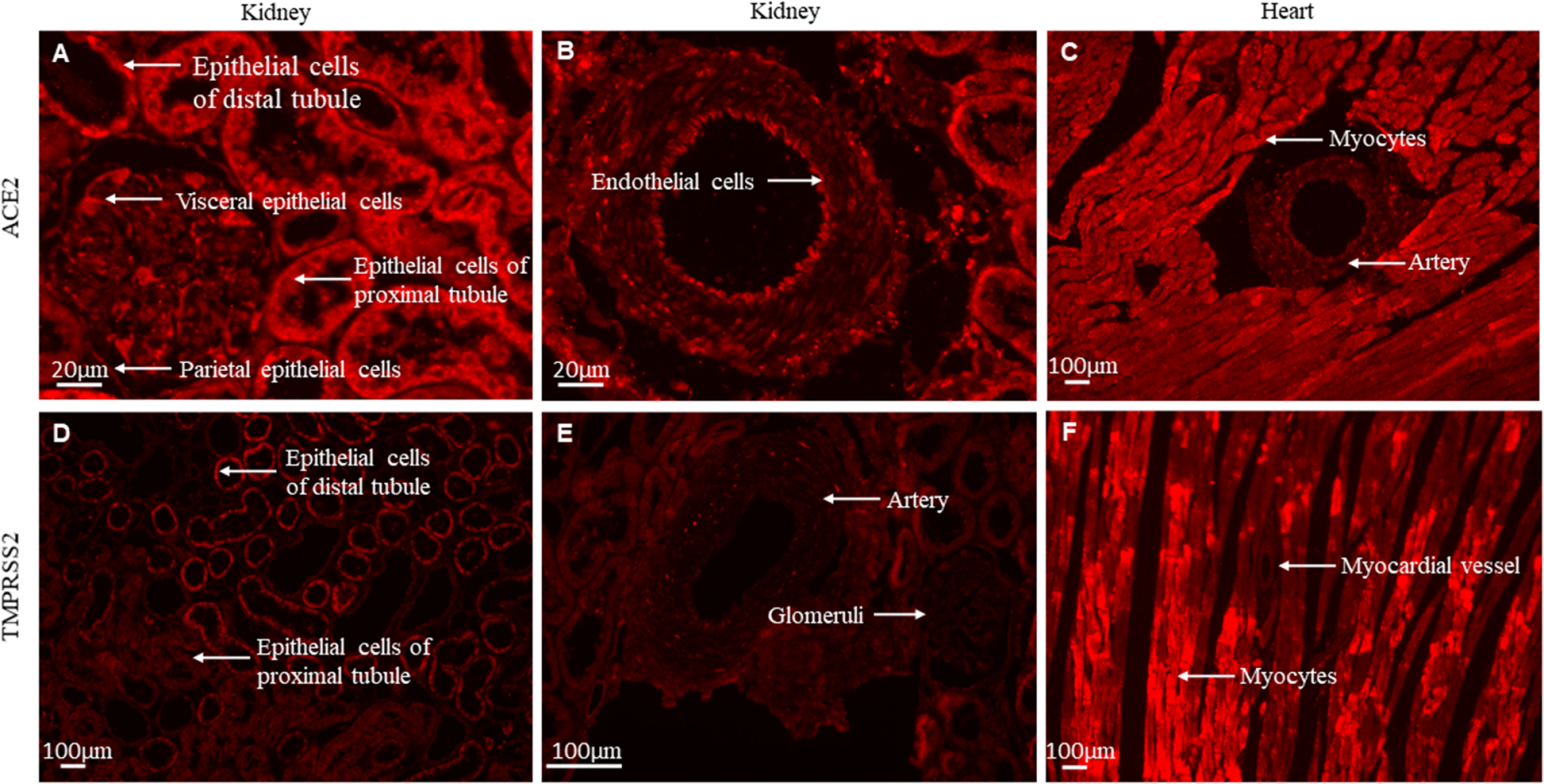
Cellular localization of renal and cardiac ACE2 and TMPRSS2 expression. Photomicrographs of immunofluorescence-stained kidney and hearts. The red color indicates ACE2 and TMPRSS2. Magnification × 20. Upper panels show immunofluorescence staining of ACE2 in the glomerulus, proximal and distal tubules **(A)**, renal arterial endothelium **(B)**, cardiac myocytes and artery **(C)**. Lower panels show immunofluorescence staining of TMPRSS2 in proximal and distal tubules **(D)**, renal artery and glomerulus **(E)**, cardiac myocytes and myocardial vessel **(F)**

We observed that in the kidney ACE2 is present in epithelial cells of the proximal tubule and distal tubule and a weak glomerular visceral ACE2 staining was observed, whereas the parietal and visceral epithelial cells were moderately positive (Fig. 3A) which was described previously [29, 30]. ACE2 is also observed in arterial endothelial cells (Fig. 3B). Moreover, ACE2 was predominantly found in tubules and a lesser extent in glomeruli. This is consistent with other studies also performed in rat kidney that found *Ace2* mRNA expression in tubules to be significantly higher expressed compared with in glomeruli [31, 32]. In the heart, ACE2 was found stronger expressed in myocytes than in arteries [33](Fig. 3C). TMPRSS2, in the kidney, was higher expressed in the distal convoluted tubule, but less expressed in the proximal tubule [34](Fig. 3D), arteries and glomeruli (Fig. 3E) whereas in the heart, TMPRSS2 is predominantly expressed in myocytes (Fig. 3F).

## Discussion

We used the ARB telmisartan, the DPP-4 inhibitor linagliptin and the SGLT2 blocker empagliflozin, in doses where we found positive pharmacodynamic action on systolic and diastolic blood pressures for all drugs under high salt diet conditions in the well-established experimental non-diabetic rat 5/6 nephrectomy. Our study shows that the induced effects on renal and cardiac mRNA and protein expression of the two key host proteins for SARS CoV-2 viral host cell entry (ACE2 and TMPRSS2) do not provide any evidence about facilitating SARS CoV-2 virus infection via the above-mentioned host receptors. The renal and cardiac gene expression level of *Ace2* was not affected either under disease conditions or under treatment conditions. Recently, it was demonstrated that the renal *Ace2* expression was not significantly altered in a STZ/high fat diet induced diabetic mouse model even if the animals were treated with ramipril (ACE inhibitor) or telmisartan [19], whereas the ACE2 protein expression was increased in this diabetic model independent of any treatment regimen. Our experimental model showed no effects on ACE2 protein expression after 5/6 nephrectomy in both the kidney and heart. Interestingly, high salt conditions led to significantly lower ACE2 level in the kidney which was normalized by linagliptin treatment, whereas the cardiac levels were unaffected. Linagliptin treatment significantly increased renal ACE2 level whereas this expression level was similar to the control groups sham and 5/6 Nx normal diet-fed rats. A recent study demonstrated that the administration of linagliptin significantly increased the ACE2 expression, which is consistent with this finding[35]. In addition, our study also revealed no significant effects on TMPRSS2 level (mRNA and protein) in the kidney which is consistent with previous finding in the experimental diabetes model [19]. However, the cardiac TMPRSS2 protein expression was significantly increased in the heart after 5/6 Nx and all drug interventions led to normalized cardiac TMPRSS2 suggesting a beneficial effect with regards to lower viral entry targets.

The discrepancy between changes in ACE2 and TMPRSS2 mRNA and protein expression was previously described in mouse and human studies [19, 36–38] indicating that the expression of ACE2 and TMPRSS2 is regulated at the post-transcriptional level. Recent studies demonstrated that post-transcriptional regulation of ACE2 can occur via microRNAs [39] or protein shedding [40]. Single-cell sequencing analysis revealed that *Ace2* is predominantly expressed in proximal tubules, whereas *Tmprss2* is predominantly expressed in the distal nephron [41, 42]. In heart tissue, Qi et al. showed that the cardiomyocytes contain 6% ACE2-expressing cells and 0.8% TMPRSS2-expressing cells [43] which might explain the absence of detectable cardiac *Tmprss2* mRNA levels in our study.

Our study revealed that only the expression of renin was affected by a more than tenfold suppressed level in placebo treated 5/6 Nx rats which has previously been described [44]. Telmisartan normalized the *Ren* mRNA level compared to linagliptin treated 5/6 Nx rats as detailed recently [45]. Also, empagliflozin restored the renin levels in 5/6 Nx rats. In a sub‐study of a double‐blind, randomized, placebo‐controlled, multicentre study (EMPA‐RESPONSE‐AHF) empagliflozin treatment was associated with a significant increase in plasma renin compared to placebo treated patients [46]. In sham kidneys, abundant expression of *Ren* mRNA was noted in the juxtaglomerular apparatus and not in the tubular epithelium whereas subtotal nephrectomy (STNx) resulted in decreased renin level based on the loss of renal mass. Moreover, altered distribution of renin gene expression was detected in the kidney of nephrectomized rats resulted by de novo renin expression in renal tubular epithelial cells with minimal or absent expression in the juxtaglomerular apparatus [47]. In perindopril-treated STNx rats, areas distant from the infarct scar demonstrated a pattern of renin gene transcription similar to that of control animals which is in line with our findings observed in telmisartan and empagliflozin treated rats.

A recently conducted comprehensive meta-analysis reported that RAAS-blocking drugs are not associated with increased risk of severe outcomes in COVID-19 patients and may further decrease all-cause mortality in COVID-19 patients[1]. Furthermore, DPP4 plays a role in SARS-CoV-2 infection as a co-receptor, and sDPP4 levels are upregulated in obesity and T2DM, possibly complicating disease outcomes, if these patients acquire COVID-19. DPP-4 inhibitors are currently investigated as a therapeutic approach preventing cardiovascular complications in COVID-19 due to their anti-inflammatory effects at the vascular level. Several clinical studies are currently under investigation which use RAAS-blocking drugs (BRACE-CORONA (NCT04364893)), gliptins (SIDIACO (NCT04365517); linagliptin trials NCT04371978 & NCT04341935) and SGLT2 inhibitors (DARE-19 (NCT04350593) in COVID-19 patients.

Our study also has limitations. First, it must be shown that our data in a rat CKD model are transferable to humans. It is also important to investigate other animal models to verify whether our observations regarding the regulation of SARS CoV-2 host factors can also be found in other CKD animal models and thus be generalized. In particular, CKD animal models with diabetes would also be of interest.

## Conclusion

Our study revealed that telmisartan, linagliptin and empagliflozin are not associated with a further increase in ACE2 and TMPRSS2 levels in kidney and heart tissue under high-salt condition compared to sham control and normal diet-fed 5/6 nephrectomy rats. The results obtained in a preclinical, experimental non-diabetic kidney failure model need confirmation in ongoing interventional clinical trials. Ongoing clinical trials with above mentioned drugs in the setting of COVID-19 will ultimately clarify their potential involvement.

## Data Availability

The datasets used and/or analyzed during the current study are available from the corresponding author on reasonable request.

## Abbreviations

ACE2: Angiotensin-converting enzyme 2
ACR: Albumin-to-creatinine ratio
ARB: Angiotensin II receptor blockers
BNP45: Brain Natriuretic Peptide-45
BW: Body weight
CKD: Chronic kidney disease
DPP-4: Dipeptidyl peptidase-4
EDTA: Ethylenediaminetetraacetic acid
ELISA: Enzyme-Linked Immunosorbent Assay
HSD: High salt-diet
ND: Normal diet
PBS-T: Phosphate-buffered saline/Tween 20
PBO: Placebo
RAAS: Renin-angiotensin-aldosterone system
SGLT2: Sodium-glucose Cotransporter-2
SARS-CoV-2: Severe acute respiratory syndrome coronavirus 2
SHR: Spontaneously hypertensive rats
TMPRSS2: Transmembrane protease, serine-subtype-2

## References

[1] Chu C, Zeng S, Hasan AA, Hocher CF, Kramer BK, Hocher B (2021). Comparison of infection risks and clinical outcomes in patients with and without SARS-CoV-2 lung infection under renin-angiotensin-aldosterone system blockade: Systematic review and meta-analysis. Br J Clin Pharmacol.

[2] Valencia I, Peiro C, Lorenzo O, Sanchez-Ferrer CF, Eckel J, Romacho T (2020). DPP4 and ACE2 in Diabetes and COVID-19: Therapeutic Targets for Cardiovascular Complications?. Front Pharmacol.

[3] Rothlin RP, Duarte M, Pelorosso FG, Nicolosi L, Salgado MV, Vetulli HM, Spitzer E (2021). Angiotensin Receptor Blockers for COVID-19: Pathophysiological and Pharmacological Considerations About Ongoing and Future Prospective Clinical Trials. Front Pharmacol.

[4] Patoulias D, Papadopoulos C, Katsimardou A, Toumpourleka M, Doumas M (2020). Sodium-Glucose Cotransporter 2 Inhibitors and Major COVID-19 Outcomes: Promising Mechanisms, Conflicting Data, and Intriguing Clinical Decisions. Diabetes Ther.

[5] Ribeiro-Oliveira A, Nogueira AI, Pereira RM, Boas WW, Dos Santos RA (2008). Simoes e Silva AC: The renin-angiotensin system and diabetes: an update. Vasc Health Risk Manag.

[6] Heurich A, Hofmann-Winkler H, Gierer S, Liepold T, Jahn O, Pohlmann S (2014). TMPRSS2 and ADAM17 cleave ACE2 differentially and only proteolysis by TMPRSS2 augments entry driven by the severe acute respiratory syndrome coronavirus spike protein. J Virol.

[7] Hoffmann M, Kleine-Weber H, Schroeder S, Kruger N, Herrler T, Erichsen S, Schiergens TS, Herrler G, Wu NH, Nitsche A (2020). SARS-CoV-2 Cell Entry Depends on ACE2 and TMPRSS2 and Is Blocked by a Clinically Proven Protease Inhibitor. Cell.

[8] Wang H, Yang P, Liu K, Guo F, Zhang Y, Zhang G, Jiang C (2008). SARS coronavirus entry into host cells through a novel clathrin- and caveolae-independent endocytic pathway. Cell Res.

[9] Kuba K, Imai Y, Penninger JM (2006). Angiotensin-converting enzyme 2 in lung diseases. Curr Opin Pharmacol.

[10] Delpino MV, Quarleri J (2020). SARS-CoV-2 Pathogenesis: Imbalance in the Renin-Angiotensin System Favors Lung Fibrosis. Front Cell Infect Microbiol.

[11] Sajuthi SP, DeFord P, Li Y, Jackson ND, Montgomery MT, Everman JL, Rios CL, Pruesse E, Nolin JD, Plender EG (2020). Type 2 and interferon inflammation regulate SARS-CoV-2 entry factor expression in the airway epithelium. Nat Commun.

[12] Chen L, Li X, Chen M, Feng Y, Xiong C (2020). The ACE2 expression in human heart indicates new potential mechanism of heart injury among patients infected with SARS-CoV-2. Cardiovasc Res.

[13] Deshotels MR, Xia H, Sriramula S, Lazartigues E, Filipeanu CM (2014). Angiotensin II mediates angiotensin converting enzyme type 2 internalization and degradation through an angiotensin II type I receptor-dependent mechanism. Hypertension.

[14] Raj VS, Mou H, Smits SL, Dekkers DH, Muller MA, Dijkman R, Muth D, Demmers JA, Zaki A, Fouchier RA (2013). Dipeptidyl peptidase 4 is a functional receptor for the emerging human coronavirus-EMC. Nature.

[15] Vankadari N, Wilce JA (2020). Emerging WuHan (COVID-19) coronavirus: glycan shield and structure prediction of spike glycoprotein and its interaction with human CD26. Emerg Microbes Infect.

[16] Ferrario CM, Jessup J, Gallagher PE, Averill DB, Brosnihan KB, Ann Tallant E, Smith RD, Chappell MC (2005). Effects of renin-angiotensin system blockade on renal angiotensin-(1–7) forming enzymes and receptors. Kidney Int.

[17] Burchill LJ, Velkoska E, Dean RG, Griggs K, Patel SK, Burrell LM (2012). Combination renin-angiotensin system blockade and angiotensin-converting enzyme 2 in experimental myocardial infarction: implications for future therapeutic directions. Clin Sci (Lond).

[18] Soler MJ, Ye M, Wysocki J, William J, Lloveras J, Batlle D (2009). Localization of ACE2 in the renal vasculature: amplification by angiotensin II type 1 receptor blockade using telmisartan. Am J Physiol Renal Physiol.

[19] Batchu SN, Kaur H, Yerra VG, Advani SL, Kabir MG, Liu Y, Klein T, Advani A (2021). Lung and Kidney ACE2 and TMPRSS2 in Renin-Angiotensin System Blocker-Treated Comorbid Diabetic Mice Mimicking Host Factors That Have Been Linked to Severe COVID-19. Diabetes.

[20] Wysocki J, Lores E, Ye M, Soler MJ, Batlle D (2020). Kidney and Lung ACE2 Expression after an ACE Inhibitor or an Ang II Receptor Blocker: Implications for COVID-19. J Am Soc Nephrol.

[21] Varagic J, Ahmad S, Brosnihan KB, Habibi J, Tilmon RD, Sowers JR, Ferrario CM (2010). Salt-induced renal injury in spontaneously hypertensive rats: effects of nebivolol. Am J Nephrol.

[22] Bernardi S, Toffoli B, Zennaro C, Tikellis C, Monticone S, Losurdo P, Bellini G, Thomas MC, Fallo F, Veglio F (2012). High-salt diet increases glomerular ACE/ACE2 ratio leading to oxidative stress and kidney damage. Nephrol Dial Transplant.

[23] Reeves PG, Nielsen FH, Fahey GC (1993). AIN-93 purified diets for laboratory rodents: final report of the American Institute of Nutrition ad hoc writing committee on the reformulation of the AIN-76A rodent diet. J Nutr.

[24] Aroor AR, Sowers JR, Bender SB, Nistala R, Garro M, Mugerfeld I, Hayden MR, Johnson MS, Salam M, Whaley-Connell A (2013). Dipeptidylpeptidase inhibition is associated with improvement in blood pressure and diastolic function in insulin-resistant male Zucker obese rats. Endocrinology.

[25] Tsuprykov O, Ando R, Reichetzeder C, von Websky K, Antonenko V, Sharkovska Y, Chaykovska L, Rahnenfuhrer J, Hasan AA, Tammen H (2016). The dipeptidyl peptidase inhibitor linagliptin and the angiotensin II receptor blocker telmisartan show renal benefit by different pathways in rats with 5/6 nephrectomy. Kidney Int.

[26] Hasan AA, von Websky K, Reichetzeder C, Tsuprykov O, Gaballa MMS, Guo J, Zeng S, Delic D, Tammen H, Klein T (2019). Mechanisms of GLP-1 receptor-independent renoprotective effects of the dipeptidyl peptidase type 4 inhibitor linagliptin in GLP-1 receptor knockout mice with 5/6 nephrectomy. Kidney Int.

[27] Delic D, Wolk K, Schmid R, Gabrielyan O, Christou D, Rieber K, Rolser M, Jakob I, Wiech F, Griesser M (2020). Integrated microRNA/mRNA expression profiling of the skin of psoriasis patients. J Dermatol Sci.

[28] Zeng S, Delic D, Chu C, Xiong Y, Luo T, Chen X, Gaballa MMS, Xue Y, Chen X, Cao Y, Hasan AA, Stadermann K, Frankenreiter S, Yin L, Krämer BK, Klein T, Hocher B. Antifibrotic effects of low dose SGLT2 Inhibition with empagliflozin in comparison to Ang II receptor blockade with telmisartan in 5/6 nephrectomised rats on high salt diet. Biomed Pharmacother. 2022;146:112606. 10.1016/j.biopha.2021.112606. 10.1016/j.biopha.2021.112606 34968924

[29] Hamming I, Timens W, Bulthuis ML, Lely AT, Navis G, van Goor H (2004). Tissue distribution of ACE2 protein, the functional receptor for SARS coronavirus. A first step in understanding SARS pathogenesis. J Pathol.

[30] Ye M, Wysocki J, William J, Soler MJ, Cokic I, Batlle D (2006). Glomerular localization and expression of Angiotensin-converting enzyme 2 and Angiotensin-converting enzyme: implications for albuminuria in diabetes. J Am Soc Nephrol.

[31] Tikellis C, Johnston CI, Forbes JM, Burns WC, Burrell LM, Risvanis J, Cooper ME (2003). Characterization of renal angiotensin-converting enzyme 2 in diabetic nephropathy. Hypertension.

[32] Li N, Zimpelmann J, Cheng K, Wilkins JA, Burns KD (2005). The role of angiotensin converting enzyme 2 in the generation of angiotensin 1–7 by rat proximal tubules. Am J Physiol Renal Physiol.

[33] Sakamoto A, Kawakami R, Kawai K, Gianatti A, Pellegrini D, Kutys R, Guo L, Mori M, Cornelissen A, Sato Y (2021). ACE2 (Angiotensin-Converting Enzyme 2) and TMPRSS2 (Transmembrane Serine Protease 2) Expression and Localization of SARS-CoV-2 Infection in the Human Heart. Arterioscler Thromb Vasc Biol.

[34] Chen Z, Hu J, Liu L, Chen R, Wang M, Xiong M, Li ZQ, Zhao Y, Li H, Guan C (2021). SARS-CoV-2 Causes Acute Kidney Injury by Directly Infecting Renal Tubules. Front Cell Dev Biol.

[35] Zhang LH, Pang XF, Bai F, Wang NP, Shah AI, McKallip RJ, Li XW, Wang X, Zhao ZQ (2015). Preservation of Glucagon-Like Peptide-1 Level Attenuates Angiotensin II-Induced Tissue Fibrosis by Altering AT1/AT 2 Receptor Expression and Angiotensin-Converting Enzyme 2 Activity in Rat Heart. Cardiovasc Drugs Ther.

[36] Wysocki J, Ye M, Soler MJ, Gurley SB, Xiao HD, Bernstein KE, Coffman TM, Chen S, Batlle D (2006). ACE and ACE2 activity in diabetic mice. Diabetes.

[37] Wijnant SRA, Jacobs M, Van Eeckhoutte HP, Lapauw B, Joos GF, Bracke KR, Brusselle GG (2020). Expression of ACE2, the SARS-CoV-2 Receptor, in Lung Tissue of Patients With Type 2 Diabetes. Diabetes.

[38] Sparks MA, South AM, Badley AD, Baker-Smith CM, Batlle D, Bozkurt B, Cattaneo R, Crowley SD, Dell'Italia LJ, Ford AL (2020). Severe Acute Respiratory Syndrome Coronavirus 2, COVID-19, and the Renin-Angiotensin System: Pressing Needs and Best Research Practices. Hypertension.

[39] Widiasta A, Sribudiani Y, Nugrahapraja H, Hilmanto D, Sekarwana N, Rachmadi D (2020). Potential role of ACE2-related microRNAs in COVID-19-associated nephropathy. Noncoding RNA Res.

[40] Palau V, Riera M, Soler MJ (2020). ADAM17 inhibition may exert a protective effect on COVID-19. Nephrol Dial Transplant.

[41] Batlle D, Soler MJ, Sparks MA, Hiremath S, South AM, Welling PA, Swaminathan S (2020). Covid, Ace2 in Cardiovascular L, Kidney Working G: Acute Kidney Injury in COVID-19: Emerging Evidence of a Distinct Pathophysiology. J Am Soc Nephrol.

[42] Dong M, Zhang J, Ma X, Tan J, Chen L, Liu S, Xin Y, Zhuang L (2020). ACE2, TMPRSS2 distribution and extrapulmonary organ injury in patients with COVID-19. Biomed Pharmacother.

[43] Qi J, Zhou Y, Hua J, Zhang L, Bian J, Liu B, Zhao Z, Jin S (2021). The scRNA-seq Expression Profiling of the Receptor ACE2 and the Cellular Protease TMPRSS2 Reveals Human Organs Susceptible to SARS-CoV-2 Infection. Int J Environ Res Public Health.

[44] Tank JE, Moe OW, Star RA, Henrich WL (1996). Differential regulation of rat glomerular and proximal tubular renin mRNA following uninephrectomy. Am J Physiol.

[45] Delic D, Wiech F, Urquhart R, Gabrielyan O, Rieber K, Rolser M, Tsuprykov O, Hasan AA, Kramer BK, Baum P (2020). Linagliptin and telmisartan induced effects on renal and urinary exosomal miRNA expression in rats with 5/6 nephrectomy. Sci Rep.

[46] Boorsma EM, Beusekamp JC, Ter Maaten JM, Figarska SM, Danser AHJ, van Veldhuisen DJ, van der Meer P, Heerspink HJL, Damman K, Voors AA (2021). Effects of empagliflozin on renal sodium and glucose handling in patients with acute heart failure. Eur J Heart Fail.

[47] Gilbert RE, Wu LL, Kelly DJ, Cox A, Wilkinson-Berka JL, Johnston CI, Cooper ME (1999). Pathological expression of renin and angiotensin II in the renal tubule after subtotal nephrectomy. Implications for the pathogenesis of tubulointerstitial fibrosis. Am J Pathol.

